# A mixed methods evaluation of the effectiveness of an oral health training program for disability care workers in Burkina Faso

**DOI:** 10.1186/s12903-023-03837-8

**Published:** 2024-01-06

**Authors:** Ave Põld, Dan Filwendé Kientega, Jocelyne Valérie Garé, Stefan Listl

**Affiliations:** 1 Institute of International Health, Charité – Universitätsmedizin Berlin, Berlin, Germany; 2 Department of Public Health, Training and Research Unit in Health Sciences, Joseph KI-ZERBO University, Ouagadougou, Burkina Faso; 3 Section for Translational Health Economics, Department for Conservative Dentistry, Heidelberg University Hospital, Heidelberg, Germany; 4 Department of Dentistry - Quality and Safety of Oral Health Care, Radboud University Medical Center, Radboud Institute for Health Sciences, Nijmegen, Netherlands

**Keywords:** Training effectiveness, Oral health, Caregivers, Disabled persons, Burkina Faso

## Abstract

**Background:**

While efforts to improve the oral health of vulnerable populations have received little attention in general, the situation of children with disabilities in low- income countries (LICs) remains especially challenging. The present study evaluated the effectiveness of an oral health training provided to disability care workers in Ouagadougou, Burkina Faso thereby contributing to closing the knowledge gap in disability research in relation to oral health in LICs.

**Methods:**

This was a single-arm pre-post study following an embedded mixed methods design using the New World Kirkpatrick training effectiveness evaluation model. For the purposes of this study, three levels of the Kirkpatrick (KP) evaluation were considered: reaction, learning and behaviour.

**Results:**

A total of 44 care workers from 6 disability centres participated in the study. Care worker post-training scores (Md = 17) were significantly higher compared to pre-training scores (Md = 13) [Wilcoxon signed-ranks test: Z= -5.53, p < .001, r = .59.] The median value for care worker confidence in applying training material in their everyday job was 7 out of 10 points (IQR = 3). At the 1-month training follow-up, 3 centres had implemented daily toothbrushing for people with disabilities.

**Conclusion:**

These findings suggest that tailored training led to an increase in care worker confidence and motivation to implement oral health activities, in knowledge about oral health and a partial implementation uptake of daily toothbrushing in disability centres. Further long-term evaluations with dental care provision in rural and urban settings are needed to lower the high oral disease burden of people with disabilities in Burkina Faso.

**Supplementary Information:**

The online version contains supplementary material available at 10.1186/s12903-023-03837-8.

## Background

Access to oral health for vulnerable and underserved populations such as children with disabilities is considered as a fundamental human right [1]. While efforts to improve the oral health of vulnerable and underserved populations have received little attention in general, the situation of children with disabilities is most challenging in low- income countries (LICs). Children with disabilities rely on their carers to monitor their oral health, yet professional care workers in LICs often lack any formal training in oral health [2]. They lack knowledge in oral diseases, in the various oral side-effects of medications patients take and in identifying the oral needs of their dependents [3].

Previous evidence on interventions to improve the oral health of people with disabilities comes mainly from high-income countries (HICs) [4]. For LICs, however, there is an absence of evidence on how the oral health of people with disabilities in being tackled. The Cochrane Oral Health Group has investigated the impact of various oral hygiene programs for people with intellectual disabilities in a systematic review indicating that training care workers of people with intellectual diseases to brush patients’ teeth improved their knowledge of oral hygiene and that a short-term impact on lowered plaque indexes was observed after training intellectually disabled patients to brush their own teeth [5]. A recent systematic review found evidence from high-income settings that oral health training interventions for carers of people with disabilities improved their knowledge, attitude, self-efficacy and behaviour towards oral health [6, 7], yet authors highlighted the need for further research in testing the effectiveness of training interventions and using assessment techniques for better understanding views of the trainees and actual oral health outcomes of people with disabilities [8]. There is also a reported need for improving the quality of evaluating health capacity building initiatives, especially ones done in low-middle income settings [9, 10].

Issues regarding the situation of persons with disabilities in Burkina Faso are scarcely researched up until now [11]. In this country where more than 6% of the population is internally displaced due to insecurity, the situation of children living with disabilities is complicated leaving 2 out of 3 disabled children without any support and care outside their family [12, 13].

Against this background, the present study aimed to evaluate the effectiveness of an oral health training programme provided to disability care workers in Ouagadougou, Burkina Faso, and thereby contribute to closing the knowledge gap in disability research in relation to oral health in LICs.

## Methods

### Setting

This study took place with care workers from six specialized centres for people with disabilities in Ouagadougou, the capital of Burkina Faso in September- November 2022. A description of each centre has been provided in Table 1.

**Table 1:** Description of disability care centres

Centre	Description	Approximate No. of residents	No. of care workers
1	Daycare centre, primary and secondary school providing specialized education for children with disabilities. The centre has medical personnel comprising a nurse and psychologist.	63	11
2	Daycare centre providing specialized education for children with disabilities. They work in two shifts dividing children into morning and evening groups. There is no medical centre, but some psychologists and motor rehabilitation service providers.	110	14
3	Daycare centre providing specialized education only in the mornings for children with disabilities. There is no medical centre, but they have partnerships with some specialists that assist in managing the children.	22	10
4	Centre that does not provide specialized education. Children visiting or residing can learn some simple jobs (gardening). There is no medical centre, but a psychologist visits each Thursday. This is the only centre where some people also reside, most just visit the centre daily.	18	11
5	This is the only facility that performs uniquely home visits. There is no specialized school or centre. They do not have a medical centre but partnerships with some medical facilities.	86	4
6	This centre does not provide specialized education for people with disabilities. This is a daycare centre for only girls with disabilities, where they learn sewing. There are no medical personnel but partnerships with some medical centres.	27	4

#### Intervention: oral health training

A 1-day oral health training workshop titled “Oral health for care workers of people with disabilities” (La santé bucco-dentaire pour le personnel encadrant des personnes vivant avec un handicap) was organised for the care workers. The workshop was facilitated by previously trained six local dental students to increase local ownership, decrease potential language barriers (French and Mooré proficiency) and cultural biases and foster future collaboration for improving the oral health of people with disabilities. The training lasted for 7 h and covered a range of topics such as anatomy of the oral cavity, oral diseases, healthy diets and oral hygiene. The last activity of the training was practical, where care workers were taught correct brushing techniques on dental models and explained the use of finger aids. The care workers filled in a written pre-test before the training and a post-test and feedback form at the end of the training day.

### Study design

This is a single-arm pre-post study following an embedded mixed methods design [14]. The rationale for embedding a qualitative component within a quantitative design derived from the need to evaluate in greater depth various factors influencing training outcomes within the study population and across different centres. The overall effectiveness of the oral health training was evaluated based on the New World Kirkpatrick training evaluation model [15]. For the purposes of this study, 3 levels of the Kirkpatrick (KP) evaluation were used: reaction (care workers’ reaction to the oral health training), learning (the degree to which the participating care workers acquired knowledge and skills of oral health via the training) and behaviour (change of oral health related on-the-job behaviour of care workers).

### Study population and recruitment

Managers of the six specialised centres for children and adolescents with disabilities were approached via invitation letters to participate in the study. Upon receiving their agreement, informed consent to participate in the study activities was gathered from all care workers in written form in the official language (French). They were presented an information sheet about the study by a local research team member and asked to sign a consent form. Enrolment to the study was voluntary and participants could withdraw from the study at any given point. Due to the small size of the target population of this study, a total population sampling method was used leading to a total of 45 care workers from 6 centres providing consent to participate in study activities [16].

### Data collection and tools

The formative phase of the study consisted of conducting qualitative observations and interviews at target sites, the results of which have been presented in an earlier publication [17]. The current paper presents results of the training implementation evaluation which comprised of collecting (a) data evaluating the short-term training outcomes (KP “Reaction” and “Learning” of Kirkpatrick) in the form of feedback forms and pre- and post-training surveys administered to care workers and (b) data evaluating the mid-term training outcomes (KP “Behaviour”) in the form of post-training observation and stakeholder surveys. All data collection tools were originally written in English and translated to French. The data collection was performed by the local dental students who also facilitated the oral health training.

#### Evaluating training outcomes (KP levels 1–3)

A unique code in the form of an acrostic was created for each study subject to de-identify data. The test subjects answered to 3 questions (“What day of the month is your birthday?”, “What is the last letter of your mother’s first name?”, “How many letters are there in your first name?”) before filling in each of the data collection sheets (pre-test, post-test, feedback form). This way we could match all 3 types of data later for the analysis phase. The individual centres were coded using the numbers 1–6 to enable centre-level analysis.

#### Data collection instruments

- Feedback form (KP Level 1- Reaction).

The first level of the Kirkpatrick evaluation measures the degree to which participants find the oral health training favourable, engaging, and relevant to their everyday work with children with disabilities (Kirkpatrick & Kirkpatrick, 2021). These components were measured at the end of the training day using paper-based training feedback forms that care workers filled in. The feedback form comprised of three sections: (a) 6 “yes/no” statements evaluating agreement about the training (b) 2 open-ended questions about the applicability of training material and necessary resources (c) a 10-point scale rating of confidence following the training.

(b) Pre-and post-test (KP Level 2- Learning).

Kirkpatrick level 2 aims to measure how well participants acquired the intended knowledge from the training but also assesses their attitude, confidence, and commitment to pick up new activities to their regular job after completing the learning day. The knowledge acquisition component was measured using a pre-and post- test consisting of single and multiple-choice questions related to oral health that were covered during the training.

The questions for the pre- and post-tests were adopted from relevant studies assessing oral health related knowledge among care workers, also children and adults in various contexts. The survey covered 5 domains: I Demography, II Oral health knowledge and behaviour [18], III Perceptions of children’s oral health [19, 20], IV Oral health related perceived knowledge and V Oral health related actual knowledge [21–24]. The instrument’s content validity was evaluated by a set of oral health experts for relevance and clarity.

Section I (four items) gathered demographic data on gender, age, education, work experience, section II (five items) was adapted from the Hiroshima University Dental Behavioural Inventory (HU-DBI) and aimed to evaluate care worker oral health behaviours [25]. Section III (three items) comprised of five-point Likert scale items taken from the Parental–Care workers Perceptions Questionnaire (P-CPQ) to evaluate how care workers perceive the children’s everyday oral health problems. Results were coded as “Never (0)”, “Once or twice (1)”, “Sometimes (2)”, “Often (3)” [20]. Section IV (3 items) evaluated care worker perceptions about how well they believe they know about oral diseases, oral hygiene, and dental trauma management, and section V (14 items) evaluated their actual knowledge about oral health including oral hygiene, diet, oral health needs of people with disabilities.

As sections I-III gathered demographic and baseline data that should not be impacted after receiving the oral health training, only sections IV and V were administered in the post-training test for analysis and comparison with pre-training test results.

(c) Post-training observation (KP Level 3- Behaviour).

Level 3 of Kirkpatrick’s training effectiveness evaluation measures the degree to which trainees apply what they learned during training to their everyday work. It comprises three components: critical behaviours, required drivers, and on-the-job learning [15]. Kirkpatrick recommends that behaviour change can be measured a minimum of 3 months after a training has been complete but due to the limited timeframe of this study, the behaviour change among care workers was measured 1 month after the training by conducting on-site observations at five disability centres.

An observational checklist was established based on literature and previous study interventions. The team conducted observations in 5 participating centres to evaluate the extent to which oral health activities and recommendations from the training had been implemented. The observational checklist covered the following areas: I Toothbrushing II Diets and III Communication with parents about oral health. Qualitative observational notes were also collected. The data collection for observations was conducted using Kobo Toolbox.

(d) Stakeholder survey.

We collected data concerning people with disabilities and their oral health in Burkina Faso via written stakeholder surveys. The survey items were reviewed for face and content validity by a group of subject matter experts from partnering universities. Individualized surveys comprising multiple choice and open-ended questions were administered to managers of the six disability centres involved in this study about available resources, training feedback and future expectations. As the data collection and training facilitation was performed by local dental students, we wanted to evaluate future opportunities and challenges dental students might encounter when working with people with disabilities and their carers by surveying a representative of the university’s dental department.

### Data analysis

#### Quantitative data

Quantitative data analysis was performed using R (version 2022.12.0 + 353). Descriptive statistical tests were performed to assess the normality of data (Shapiro-Wilk test with a significance level of p < .05), followed by the frequencies, percentages and central tendencies of demographic variables, oral health behaviours, perceptions, pre-and post-test scores. For analysing differences in pre-and post-test scores of care workers, descriptive statistics were used where ordinal and nominal data was described as frequencies (n) and percentages (%); and discrete and continuous data using means and standard deviations (µ ± SD).

Inferential statistics were performed where pre-and post-test scores for study participants were compared using the paired Wilcoxon signed ranks test with a significance level of 0.05 at a target power of 0.95 and an expected effect size of 0.52 (calculated using G*Power 3.1) [26]. Gain score analysis was performed to understand gains in test scores after completing the training. Spearman’s rank correlation was computed to assess the association between care worker HUDBI score and gain score. Simple linear regression was used to test if pre-training test scores (knowledge) significantly predicted the HU-DBI score (behaviour).

#### Qualitative data

Feedback forms, observation notes and stakeholder surveys were coded and analysed using MAXQDA 2022 Analytics Pro version 22.4 [27]. All qualitative data was first translated from French to English for analysis. The resulting transcripts were analysed using a thematic analysis approach to generate initial codes and define, search for and review themes [28, 29].

## Results

### Demography

**Fig. 1:**
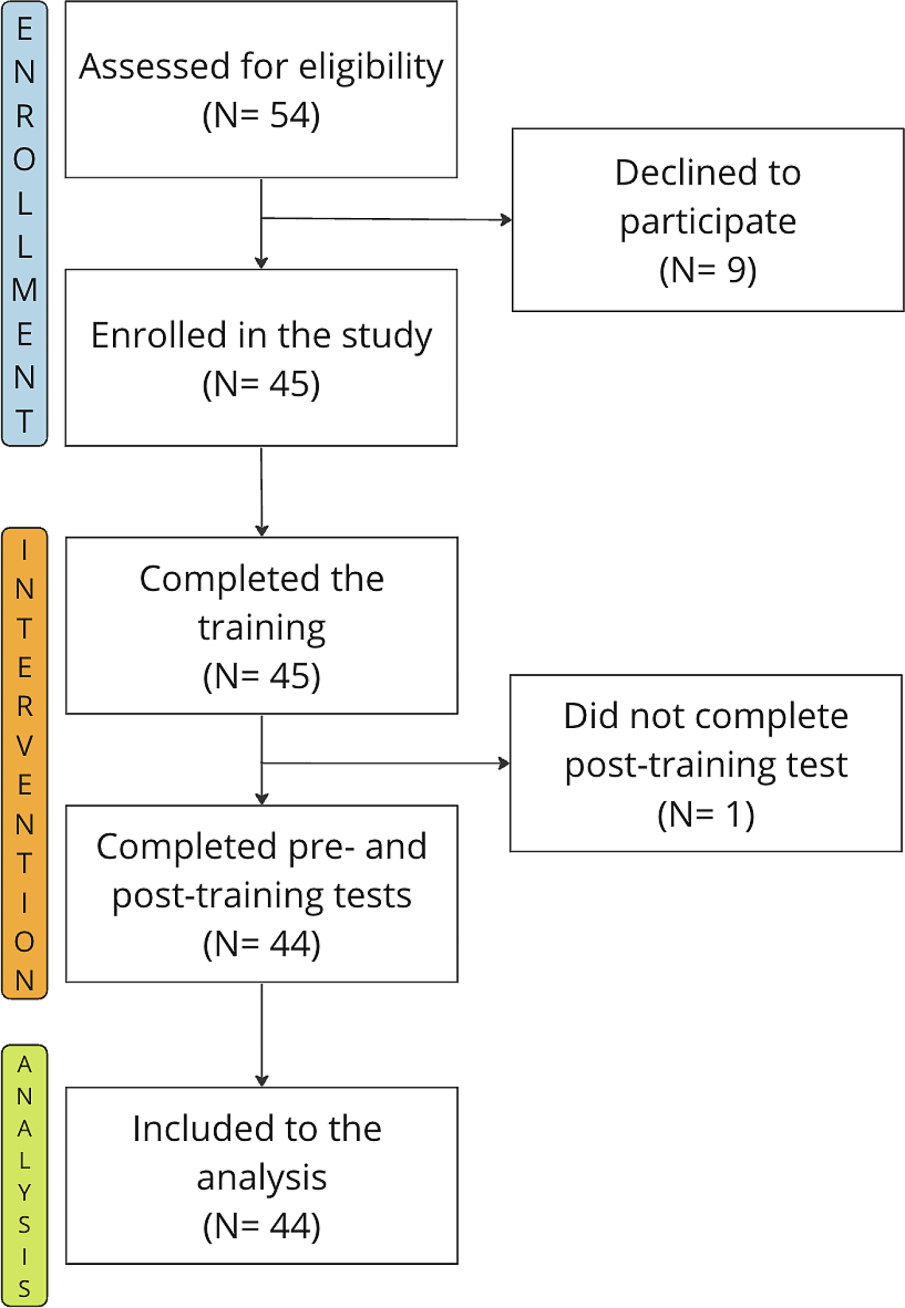
Participant flowchart

A total of 44 care workers from 6 disability centres were included in the analysis, the participant flowchart is presented in Fig. 1.

77.3% of study participants were female and 22.7% male. Most participants were 35–44 years old (40.9%), 29.5% were 45–59 years, 25.0% 18–34 years and 4.5% 60–64 years old. The majority of participants had completed secondary education (65.9%). Most care workers (34.1%) had a short working experience of 1–5 years, 25.0% had worked for 6–10 years, 27.3% for 11–20 years and just 14.0% for 20 or more years as care workers. 7 out of 8 care workers with a university education had worked 1–5 years as care workers. The distribution of care workers from the 6 centres was quite unequal ranging from two centres with just 2 care workers and the biggest centre with 14 care workers participating in the training. The demographic data of study participants are presented in Table 2.

**Table 2:** Demographics of care workers participating in the study

Variable	Frequency	Percentage
**Gender**		
Female	34	77.3%
Male	10	22.7%
**Age**		
18–34	11	25.0%
35–44	18	40.9%
45–59	13	29.5%
60–64	2	4.5%
**Education**		
Primary education	7	15.9%
Secondary education	29	65.9%
University	8	18.2%
**Working experience**		
1–5 years	15	34.1%
6–10 years	11	25.0%
11–20 years	12	27.3%
20 years or more	6	13.6%
**Centres**		
1	11	25.0%
2	14	31.8%
3	6	13.6%
4	9	20.5%
5	2	4.5%
6	2	4.5%

### Kirkpatrick evaluation level 1: reaction

#### Training feedback

A detailed overview of training feedback has been presented in Table 3. For the first section, the lowest level of agreement (90.9% of respondents) was for the statement “I will start immediately using what I have learned today in my everyday work”. When asked to explain in the second section what specifically trainees will be able to apply on their job, 52.3% of all respondents mentioned improving the oral hygiene and implementing toothbrushing with the disabled children, 18.2% mentioned improving oral hygiene and diets or just improving the everyday diet of their dependents. In response to what assistance or resources will trainees need to successfully apply what they learned on the job, 65.9% responded that they need toothbrushing materials such as toothbrushes and -paste, 20.5% would need in addition to materials also additional coaching. The need for dental check-ups for the children and additional staff to help with oral health activities, were also noted.

The last survey section asked trainees to rate on a 10-point scale their confidence in applying what they learned during the training to their everyday job. The median value across all participants was 7 (IQR = 3).

**Table 3:** Results from the trainee feedback form (Kirkpatrick Level 1)

**Kirkpatrick Level 1: Reaction. Trainee feedback form**					
**Agreement of care workers with the following statements:**				**Responses N (%)**	
				**“Yes”**	**“No”**
I was engaged with the training activities.				44 (100%)	0
The activities and exercises helped me to learn.				44 (100%)	0
I was given enough opportunities to practise what I was learning.				42 (95.5%)	2 (4.5%)
I will be able to immediately use what I have learned.				44 (100%)	0
I will start immediately using what I have learned today in my everyday work.				40 (90.9%)	4 (9.1%)
I would recommend this training to my colleagues.				44 (100%)	0
**Responses to open-ended questions:**					**Responses** **N (%)**
**From what you learned, what will you be able to apply on your job?**					
Oral hygiene/toothbrushing					23 (52.3%)
Oral hygiene and/or improving diet					8 (18.2%)
General confirmation of starting to apply what they learned in their job					11 (25.0%)
Other					2 (4.5%)
**What assistance or resources will you need to successfully apply what you learned on the job?**					
Materials (toothbrushes, toothpaste)					29 (65.9%)
Materials and/or additional coaching and support					9 (20.5%)
Professional dental check-up for children					2 (4.5%)
Additional staff to support					2 (4.5%)
Other (additional staff, separate room for materials)					2 (4.5%)
**How confident are you that you will be able to apply what you have learned back on the job?**					
**Min.**	**IQR 1**	**Median**	**Mean**	**IQR 3**	**Max.**
0.0	6.0	7.0	6.9	9.0	10.0

### Kirkpatrick evaluation level 2: learning

#### Pre- and post-test comparison

Non-parametric tests were used in the analysis as post-training test scores departed significantly from normality according to the Shapiro-Wilk test (W = 0.587, p < .001). The median pre-test score for all participants was 13 (IQR = 4) and it increased to 17 (IQR = 1) after completing the 1-day oral health training. A Wilcoxon signed-ranks test indicated that care worker post-training scores (Md = 17) significantly increased compared to pre-training scores (Md = 13), Z= -5.53, p < .001, r = .59, thus exceeding the expected effect size of 0.52 and achieving a power of 0.98, slightly exceeding the target power of 0.95. Significant results were consistent for both genders: (a) female care workers’ (n = 34) pre- and post-test scores, Z= -4.9, p < .001, r = .60 and (b) male care workers’ (n = 10) pre-and post-test scores, Z= -2.77, p < .05, r = .62.

No statistically significant correlation was found between pre-and post-test scores using the Spearman’s rank correlation, r [42] = 0.26, p = .09. The median gain of scores for all care workers were 3.0 points (IQR = 3). There were no statistically significant differences in gain scores by demographic groups.

#### Oral health knowledge and behaviour (HU-DBI)

A correlation was found between HU-DBI scores and pre-test scores, r [42] = 0.318, p = .035. Simple linear regression was used to predict if pre-training test scores significantly predicted HU-DBI scores. The fitted regression model: HU-DBI score = 0.518 + 0.19 (pre-training test score), R^2^ = 0.114, F [1, 42] = 6.542, p = .014. The histogram of standardised residuals indicated that the data contained approximately normally distributed errors, as did the normal Q-Q plot of standardised residuals. The F test for lack of fit test was insignificant, F [9, 33] = 0.71, p > .5 showing no obvious violation of the linearity assumption. Therefore, it was found that pre-training test scores significantly predicted HU-DBI results, β = 0.19, p = .014. No significant difference between female and male care worker HU-DBI scores was found (p = .33).

#### Perceptions of children’s oral health (likert items)

**Fig. 2:**
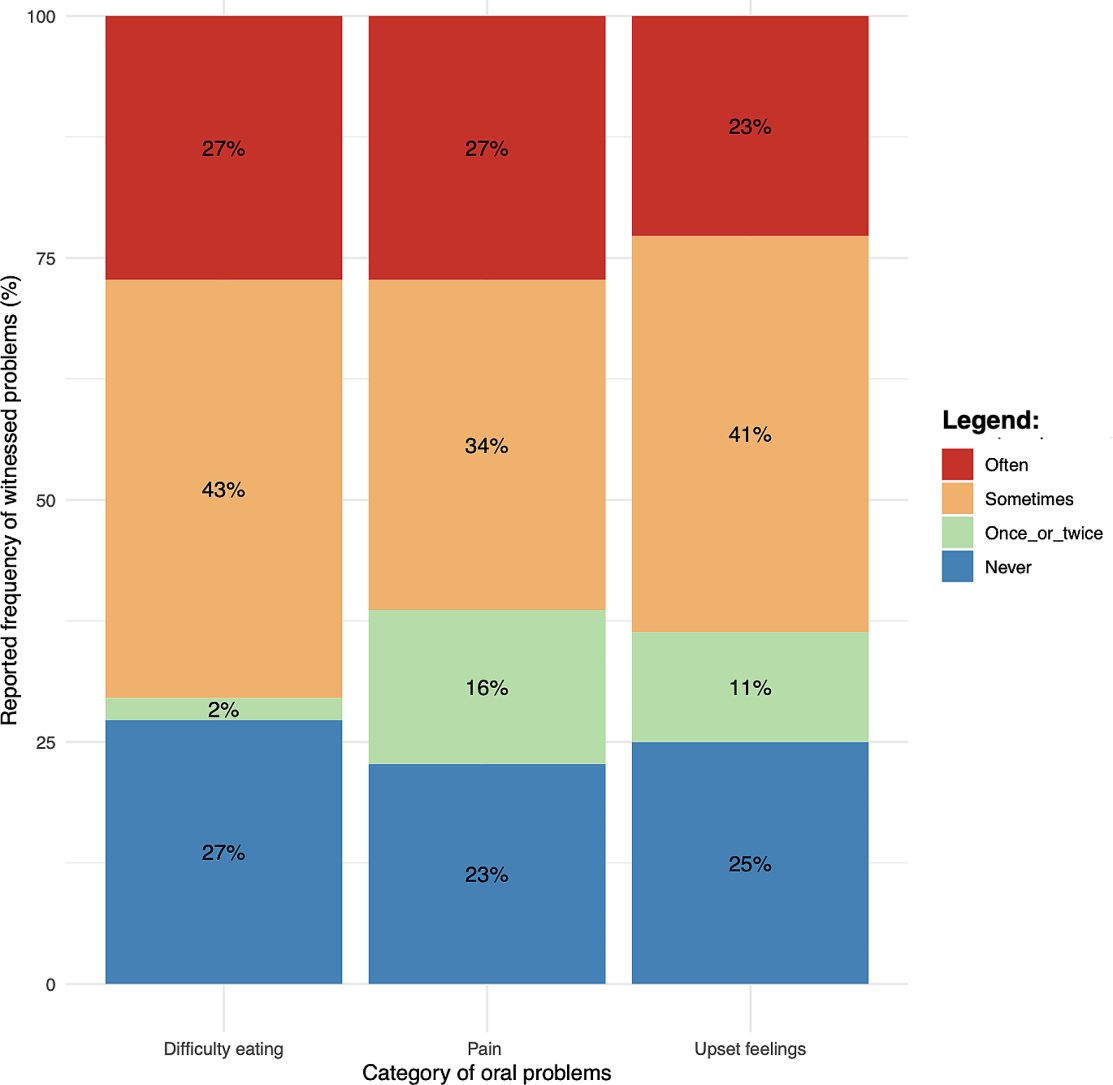
Likert scale items about the perceived oral health status of children

The median response for all questions evaluating care worker awareness of children’s oral problems was 2 (Sometimes) (Fig. 2). For each item, more than 60% of care workers responded “sometimes” or “often” showing that they have noticed on several occasions that children suffer due to problems with their teeth and oral region. In centre 4 the median value for the question, “During the last 3 months, how often have children in your establishment had: Difficulty biting or chewing foods such as fresh apple, corn on the cob or firm meat?” was highest- 3 (Often). No correlation was found between HUDBI scores and Likert scores.

#### Oral health related perceived knowledge

The Wilcoxon signed rank test indicated that care workers significantly evaluated their perceived knowledge about oral health as higher after completing the training (Z= -5.3; p < .001), r = .57.

No significant correlation was found between HU-DBI scores and pre-training perceived knowledge scores. No significant correlation was found between pre-training perceived knowledge scores and Likert items. A summary of care worker perceived knowledge on various oral health topics before and after the training is presented in Table 4.

**Table 4:** Summary table of oral health related perceived knowledge per item

	Oral disease	Oral hygiene	Trauma
**Oral health related perceived knowledge (pre-training)**			
Yes (1)	23 (52.3%)	28 (63.6%)	11 (25.0%)
No (0)	21 (47.7%)	16 (36.4%)	33 (75.0%)
**Oral health related perceived knowledge (post-training)**			
Yes (1)	43 (97.7%)	44 (100%)	43 (97.7%)
No (0)	1 (2.3%)	0	1 (2.3%)

### Kirkpatrick evaluation level 3: behaviour

The post-training observations revealed that care workers from 3 centres had started to supervise regular toothbrushing for the people with disabilities during this 1-month period following the training. One of the implementing centres was centre 4 whose care workers had previously reported most problems for children having difficulty eating due to oral problems. Care workers in one of the centres that had not yet implemented toothbrushing were observed talking to parents about the importance of proper oral hygiene for their children.

In the pre-training observation, the main harmful dietary patterns found were the consumption of sugary snacks and drinks children often brought with them from home. It is difficult to assess the full extent to which diets were modified following the training, but we witnessed during the follow-up observation that in all centres the main drink was water and that only in one centre were sweets still handed out as snacks. There were still some centres where sugar-sweetened beverages and fruit juice were consumed, also various snacks that parents had packed for their children. During the qualitative interviews conducted with care workers at the start of this project we also learned that they sometimes use food as a reinforcer for motivating children [17]. This was not observed during the post-training data collection, but that might rather be due to the short duration of the observations. A summary of the post-training observation is presented in Table A1.

#### Centre coordinators

All 6 centres receive material (vehicles, computers, office furniture, construction work), financial (donations, salaries) and technical (human resources) support from their partners. All the coordinators explained that primarily they look forward to additional financial and material (e.g., computers, medical devices for early detection, etc.) support and three coordinators added that trainings and capacity-building are also needed.

When asked for feedback about the oral health training, all centre coordinators said that it was helpful to their staff as they learned new information about oral health, gained competencies and technical skills for brushing teeth and for informing parents about the importance of good oral health. Four coordinators were looking forward to the relaunch or continuation of this oral health project, two would like to receive trainings on other health-related topics and two coordinators also highlighted the need for dental screening for the children in their centres.

#### Representative of the university’s dental faculty

Currently there is a theoretical program titled “Oral pathologies and their management among disabled children” for 5th year dental students. The faculty is interested in providing students more opportunities to work with people with disabilities. As a suggestion, oral health training and awareness sessions for the personnel supervising the disabled people (parents and social educators) could be conducted by dental students covering oral diseases and good oral hygiene practices for the disabled people. Another way of including dental students could also be through research opportunities on topics related to the oral health of people with disabilities.

When asked about the biggest challenges in enabling dental students to gain more experiences with disabled people, the faculty member mentioned the need to overcome stereotypes that people with disabilities are difficult to care for and to grow empathy towards people with disabilities. It is also important to train personnel working with disabled people about oral health so they would be more aware and skilful.

## Discussion

The overall aim of delivering an oral health training to care workers of people with disabilities and assessing its outcomes was achieved during our project. Some interesting findings from the study included an overall significant improvement of care worker test scores following the oral health training, pre-training test scores predicting the HU-DBI results, highly positive feedback towards the training, and partial implementation of toothbrushing one month following training completion.

Across the study population, the median improvement in test scores was 17.6% (median gain of 3 points), which indicates that contrary to what we presumed, most care workers had previous knowledge about basic oral health concepts. The carers’ baseline oral health knowledge did not impact the learning effect as no correlation between pre- and post-training test scores was found. The post-training test median was 17 (IQR = 1), showing that most care workers scored the maximum possible following the training day, reflecting a gain in knowledge, the relative simplicity of the test and the test evaluating content presented during the training.

Our results indicated that care workers with greater knowledge about oral health at baseline scored higher in items evaluating personal oral health behaviours (HU-DBI). This finding supports the importance of building knowledge about oral health for inducing behaviour change among care workers [30]. Trainees reported in their feedback that the oral health training increased their confidence and positive attitudes for implementing oral health to their everyday job, factors considered essential for lifelong learning [31]. The overall highly positive training feedback, ranging from 90,9 to 100% agreement for various statements, must be interpreted with caution as Burkina Faso is a high context culture with a hierarchical and collectivistic society based on Hofstede’s framework [32]. This may play a significant role in care workers’ willingness to offer constructive feedback to training facilitators [33]. Avoiding giving direct negative feedback about the training might be due to it being impolite and potentially overly critical in societies like Burkina Faso. This may lead to challenges in understanding how care workers truly perceived the training and what could have been done better.

The perceived impact oral diseases have on the well-being of people with disabilities showed that more than 60% of care workers witness sometimes or often the impacts of bad oral health on their dependents. Several care workers and coordinators mentioned that they see a need for dental check-ups to be organised for the disabled people in their centres. Organizing regularly such oral health trainings could enhance care workers’ abilities to identify oral issues, actively work towards reducing dental problems among persons with disabilities, and educate their parents about the importance of oral care and encourage them to seek timely dental treatment [34–36].

The final level of our Kirkpatrick evaluation targeted toothbrushing implementation one month after the completion of oral health trainings. We observed 3 centres conducting daily toothbrushing despite all care workers having stated during formative interviews that after the training they intend to start with regular toothbrushing [17]. There are multiple potential reasons for why all centres had not implemented brushing. Based on literature about barriers and enablers of sustainable school-based toothbrushing, organisational, staff-, parent- and child-level factors come into play [37]. Most relevant to the context of this study might be a lack of time to introduce toothbrushing as a regular daily activity with children, a lack of staff to supervise brushing (also mentioned in training feedback forms) and a lack of staff buy-in to start taking care of the oral health of their dependents [38–40]. Understanding these underlying factors would need follow-up evaluations to be conducted in combination with implementation support activities. We identified in our stakeholder survey an interest from the university’s dental department to increase opportunities for their students to work with people with disabilities. The local universities could carry a leading role in conducting these follow-up evaluations by further engaging dental students in training and research activities where they can interact with disabled individuals for improving their oral health.

Prevention alone will not solve the oral health problems of people with disabilities in Burkina Faso. Although the country has a law on the protection and promotion of the rights of persons with disabilities that states that consultations, care, medicines and hospitalization in public health structures are free for people with disabilities, it has failed to improve access to dental care for those individuals [41]. Previous studies conducted in high-income settings have highlighted various barriers to seeking dental care for people with disabilities including financial barriers, issues with transportation, anxiety and the fear of care workers themselves in seeking treatment [42–44]. It would be interesting to explore such barriers to seeking dental care in a context where care workers and potentially parents as well are aware of the oral problems of their dependents yet fail to seek and provide the necessary dental care for them. Further long-term implementation research is crucial to translate knowledge about the oral health status of people with disabilities in Burkina Faso into sustainable action towards improving their health and wellbeing.

**Limitations**.

There are some limitations to this study as only six centres for people with disabilities from the capital Ouagadougou were included thus potentially omitting rural perspectives. The data collection instruments (pre- and post-training test, feedback forms) were not piloted with a subset of care workers before the study. The post-training observation was not conducted in one of the participating centres as its care workers only perform house visits and observing them at people’s homes would have been outside of the scope of this research. Although we managed to do a follow-up observation 1 month after the training, this was a very short period for drawing conclusions and further follow-up coupled with in-depth interviews 3, 6 and 12 months later would be useful to evaluate and understand the enablers and restrains of implementing oral health activities in each centre.

## Conclusion

This study identified an increase in care worker confidence and motivation to implement oral health activities, a significant increase in knowledge about oral health and partial implementation uptake of daily toothbrushing by participating centres for disabilities in Ouagadougou one month after the training completion. There is a great need to conduct follow-up implementation research focusing on sustainability and long-term outcomes of oral health training activities in both urban and rural settings. Education and skill training must be coupled with referral opportunities for dental care to lower the oral disease burden of people with disabilities living in low-income countries such as Burkina Faso.

### Electronic supplementary material

Below is the link to the electronic supplementary material.

Supplementary Material 1

## Data Availability

The data that support the findings of this study are available on request from the corresponding author.

## Abbreviations

HIC: high-income country
HU-DBI: Hiroshima University Dental Behavioural Inventory
LIC: low-income country
KP: Kirkpatrick
P-CPQ: Parental–Caregivers Perceptions Questionnaire
