# Supplementary material for: A mixed methods evaluation of the effectiveness of an oral health training program for disability care workers in Burkina Faso
Source: BMC Oral Health. 2024 Jan 6;24:33. doi: 10.1186/s12903-023-03837-8 (PMC10771690; doi:10.1186/s12903-023-03837-8)
Supplement: Supplementary file 1 — Supplementary Material 1 [file 12903_2023_3837_MOESM1_ESM.docx]

**Table A1. Summary table of post-training observation checklist items and notes.**

| **Checklist items** | **Centers (YES)** | **Centers (NO)** |
| --- | --- | --- |
| Number of centres where toothbrushing was conducted | 3 | 2 |
|  | *“Children are mostly motivated to brush. They hold the brushes themselves and try to make the brushing motion.”*  *“The children brushed themselves, but under the supervision of the supervisors”* | *“After the snack at 10am, there was no toothbrushing session. The staff was more preoccupied with getting the children back to the various classrooms to continue the day's activities.”* |
| Number of centres where caregivers handed out sweets as snacks. | 1 | 4 |
|  | *“The children had a snack of buttered bread, local bissap juice, some children drank Fanta and kalippo juice (cocktail juice) provided by their parents.”* | *“Children are sitting on mats and others on benches. Each one with its own dish. All the children manage to feed themselves except one. There are no sweets. Everyone drinks water.”* |
| Number of centres where children mostly drank water throughout the day | 5 | 0 |
| Number of centres where caregivers spoke to the children's parents about oral health. | 1 | 4 |
|  | *“He [the coordinator] took the opportunity to introduce himself and explain the framework of the project [to the parents] and inform them of the importance of oral hygiene for children by inviting them to conduct regular toothbrushing.”* |  |
| Summaries from observations in different centres | *“There are children who brush themselves and others who need help but overall, it went well.”*  *“The supervisors show an interest in the brushing program. They are working together to get the activity done as quickly as possible. We have information that they also performed the brushing yesterday.”*  *“On the whole it's going well, the children have brushed their own teeth, they are all cooperative and considering the state of their mouths we can say that there is improvement.”* | *“The conclusion is that the staff says that they emphasize the motivation of parents to perform the brushing of children. But honestly, they do not know the sincerity of the parents if they do it or not. So, brushing did not take place during our observation. Toothbrushes are always stored in the classrooms.”*  *“We found that the supervisors follow their usual program. The brushing program was not integrated.”* |
